# Apatinib plus chemotherapy for non-metastatic osteosarcoma: a retrospective cohort study

**DOI:** 10.3389/fonc.2023.1227461

**Published:** 2023-11-13

**Authors:** Jiaqiang Wang, Fan Zhang, Shuping Dong, Yang Yang, Fangfang Gao, Guancong Liu, Peng Zhang, Xin Wang, Xinhui Du, Zhichao Tian

**Affiliations:** ^1^Department of Orthopedics, The Affiliated Cancer Hospital of Zhengzhou University and Henan Cancer Hospital, Zhengzhou, Henan, China; ^2^Modern Educational Technology Center, Henan University of Economics and Law, Zhengzhou, Henan, China; ^3^Department of Medical Oncology, The Affiliated Cancer Hospital of Zhengzhou University and Henan Cancer Hospital, Zhengzhou, Henan, China

**Keywords:** apatinib, doxorubicin, cisplatin, osteosarcoma, neoadjuvant therapy, adverse events

## Abstract

**Background:**

Effective adjuvant therapy for osteosarcoma is necessary for improved outcomes. Previous studies demonstrated that apatinib plus doxorubicin-based chemotherapy may improve the efficacy of neoadjuvant therapy. This study aimed to clarify the effectiveness and safety of apatinib plus doxorubicin and cisplatin (AP) as neoadjuvant therapy for osteosarcoma.

**Methods:**

The clinical data of osteosarcoma patients who underwent neoadjuvant therapy and surgery between August 2016 and April 2022 were retrospectively collected and analyzed. Patients were divided into two groups: the apatinib plus AP (apatinib + AP) group and the methotrexate, doxorubicin, and cisplatin (MAP) group.

**Results:**

This study included 42 patients with nonmetastatic osteosarcoma (19 and 23 patients in the apatinib + AP and MAP groups, respectively). The 1- and 2-year disease-free survival rates in the apatinib + AP group were higher than those in the MAP group, but the difference was not significant (P=0.165 and 0.283, respectively). Some adverse events were significantly more common in the apatinib + AP group than in the MAP group, including oral mucositis (grades 3 and 4) (52.6% vs. 17.4%, respectively, P=0.023), limb edema (47.4% vs. 17.4%, respectively, P=0.049), hand-foot syndrome (31.6% vs. 0%, respectively, P=0.005), proteinuria (26.3% vs. 0%, respectively, P=0.014), hypertension (21.1% vs. 0%, respectively, P=0.035), and hypothyroidism (21.1% vs. 0%, respectively, P=0.035). No drug-related deaths occurred. There was no statistically significant difference in the incidence of postoperative complications between the groups (P>0.05).

**Conclusion:**

The present study suggests that apatinib + AP may be a promising candidate for neoadjuvant therapy for osteosarcoma, warranting further validation in prospective randomized controlled clinical trials with long-term follow-up.

## 1 Introduction

Osteosarcoma is one of the most common osteogenic malignancies. There are 2,000–3,000 newly diagnosed cases annually in China (1, 2). The standard treatment for non-metastatic osteosarcoma is preoperative chemotherapy (neoadjuvant chemotherapy), surgery, and postoperative adjuvant chemotherapy (2–4). The purpose of neoadjuvant chemotherapy is to 1) eliminate small metastases that may exist; 2) shrink the tumor, increase the rate of limb salvage, and reduce the recurrence rate of osteosarcoma; and 3) determine the effect of chemotherapy to facilitate the formulation of plans for postoperative chemotherapy, thus improving the curative effect (5, 6). The drug regimen used as neoadjuvant chemotherapy has been studied repeatedly and in detail. The currently recognized regimen is a combination of methotrexate, adriamycin (doxorubicin), and cisplatin (MAP), or doxorubicin and cisplatin (AP) (6, 7). Approximately 50% of patients initially diagnosed with non-metastatic osteosarcoma develop metastasis and ultimately do not survive, even after receiving neoadjuvant chemotherapy (6, 8, 9). Therefore, new therapeutic drugs and methods are urgently required.

Apatinib was marketed in 2014 in China as the first domestically produced multi-target tyrosine kinase inhibitor (TKI) (10). Targets that are inhibited by apatinib include VEGFR1, VEGFR2, c-RET, c-KIT, and c-SRC (11, 12). Several studies have demonstrated that apatinib can inhibit the proliferation, invasion, and migration of osteosarcoma cells *in vitro* (13–15) and is effective in treating patients with advanced osteosarcoma (16).

Our previous study has demonstrated that apatinib ameliorates doxorubicin-induced migration and cancer stemness of osteosarcoma cells (17). This suggests that apatinib combined with neoadjuvant chemotherapy may achieve better outcomes in non-metastatic osteosarcoma than the current standard regimen. However, no reports have confirmed this. Some of our patients with osteosarcoma were treated with apatinib plus neoadjuvant chemotherapy in clinical practice. In the present study, we retrospectively collected and analyzed the clinical data of these patients and summarized the effectiveness and safety of this treatment regimen.

## 2 Materials and methods

### 2.1 Patient

All osteosarcoma patients included in this retrospective study were treated between August 2016 and April 2022. This study was approved by the Institutional Review Board of Henan Cancer Hospital and was conducted in accordance with the guidelines and principles of the Declaration of Helsinki. All patients included in the present study met the following criteria: 1) pathologically confirmed osteosarcoma; 2) no evidence of distant metastasis; 3) received apatinib + AP or MAP neoadjuvant and postoperative adjuvant therapy; 4) underwent limb salvage surgery or amputation.

### 2.2 Treatment protocol

Based on the different drugs received, patients were divided into two groups: the apatinib + AP group and the MAP group. In the MAP group, patients received preoperative chemotherapy comprising two 5-week cycles of doxorubicin 37.5 mg/m^2^/day for 2 days, cisplatin 60 mg/m^2^/day for 2 days, and methotrexate 12 g/m^2^ (18). Surgery was scheduled after two cycles of preoperative chemotherapy. The patients received another four cycles of treatment postoperatively, similar to preoperative chemotherapy.

Some patients chose to receive apatinib plus AP treatment (apatinib + AP group). In this group, all patients received preoperative therapy consisting of cisplatin 60 mg/m^2^ and doxorubicin 37.5 mg/m^2^ per day on days 1−2. Each patient received six cycles of chemotherapy, which were repeated every 3 weeks. Surgery was scheduled after two cycles of chemotherapy. Postoperatively, the patients received another four cycles of treatment, similar to preoperative chemotherapy. Patients in parallel received 500 mg (those with body surface area [BSA] ≥1.5 m^2^) or 250 mg (those with BSA <1.5 m^2^) apatinib per day, starting on day 3. Apatinib was interrupted during chemotherapy and interrupted for 2 weeks postoperatively and then continued until 1 year postoperatively.

Adverse events (AEs) were determined according to the Common Terminology Criteria for Adverse Events version 4.0. For patients who could not tolerate AEs, the dose of apatinib was reduced to 250 mg/day or 125 mg/day.

Limb salvage surgery or amputation was performed after two cycles of preoperative therapy. All surgeries were aimed at achieving complete resection of the primary lesion. Apatinib was interrupted for 2 weeks postoperatively and then continued until 1 year postoperatively.

### 2.3 Evaluation

The Response Evaluation Criteria in Solid Tumors (version 1.1) was used to evaluate the effectiveness of neoadjuvant treatment. Tumor responses were categorized as complete response (CR), partial response (PR), stable disease (SD), or progressive disease. The disease control rate (DCR) was defined as the sum of the rates of CR, PR and SD. The differences in alkaline phosphatase (ALP) serum level changes post-neoadjuvant therapy, tumor cell necrosis rate, and 1- and 2-year disease-free survival (DFS) rates between the two groups were evaluated. Tumor responses were evaluated according to the response evaluation criteria in solid tumors (version 1.1). DFS was defined as the time from the surgery to the first occurrence of signs of recurrence or metastasis. The between-group rates of drug-related AEs and surgery-related complications were compared. Surgery-related complications were graded using the Clavien–Dindo grading system.

### 2.4 Statistical analysis

Quantitative variables are presented as numerical values (percentages), medians (ranges), or medians (interquartile range). Categorical data were compared using Fisher’s exact test, and continuous variables were compared using Student’s t-test. Progression-free survival was estimated using the Kaplan–Meier method. The univariate Cox proportional hazards model was used to analyze the relationship between clinicopathological parameters and DFS. Statistical analyses were performed using SPSS (version 21.0; IBM, Armonk, NY, USA). All statistical analyses were two-sided, and a P-value <0.05 was considered statistically significant.

## 3 Results

### 3.1 Patient characteristics

A total of 42 osteosarcoma patients met the eligibility criteria for this study, with 19 and 23 patients included in the apatinib + AP and MAP groups, respectively. The median follow-up period was 28 (9–50) and 22 (9–42) months for the apatinib + AP and MAP groups, respectively.

The baseline characteristics of the patients are presented in **Table 1**. All patients in both groups were younger than 30 years of age. The median ages of the patients at diagnosis were 18.0 (13.0–21.0) and 18.0 (14.5–20.5) years in the apatinib + AP and MAP groups, respectively. All patients in both groups were Enneking Stage II at the time of treatment initiation. The primary lesions were most commonly located in the long bones of the extremities. The diameters of the primary lesions in most patients in the two groups were >10 cm. The pre-neoadjuvant chemotherapy ALP serum level was >200 U/L in more than half of the patients in both groups. There were no significant differences between the groups in baseline characteristics (P>0.05, **Table 1**).

**Table 1:** Patient characteristics by treatment group.

Characteristics	Apatinib + AP group (n = 19)	MAP group (n = 23)	P-value
Sex			1.000
Male	10 (52.63%)	13 (56.52%)	
Female	9 (47.37%)	10 (43.48%)	
Median age (years)	17.26 ± 5.17	17.57 ± 5.13	0.851
ECOG PS			0.763
0	11 (57.89%)	12 (52.17%)	
1	8 (42.11%)	11 (47.83%)	
Enneking stage grade			1.000
IIA	8 (42.11%)	11 (47.83%)	
IIB	11 (57.89%)	12 (52.17%)	
Primary site			0.978
Femur	6 (31.58%)	8 (34.78%)	
Axial skeleton	2 (10.53%)	3 (13.04%)	
Tibia	5 (26.32%)	6 (26.09%)	
Humerus	3 (15.79%)	4 (17.39%)	
Fibula	1 (5.26%)	0 (0.00%)	
Radial	1 (5.26%)	0 (0.00%)	
Other	1 (5.26%)	2 (8.70%)	
Histologic subtypes			1.000
Conventional	16 (84.21%)	20 (85.96%)	
Small cell	2 (10.53%	1 (4.35%)	
Telangiectatic	1 (5.26%)	2 (8.70%)	
Tumor size			0.748
Small (<10 cm)	7 (36.84%)	7 (30.43%)	
Large (≥10 cm)	12 (63.16%)	16 (69.57%)	
Pre-neoadjuvant chemotherapy ALP serum level (U/L)			1.000
<200	9 (47.37%)	11 (47.83%)	
≥200	10 (52.63%)	12 (52.17%)	

Data are presented as counts (percentages) or means ± standard deviations.

AP, doxorubicin-cisplatin chemotherapy; MAP, methotrexate-doxorubicin-cisplatin chemotherapy; ECOG PS, Eastern Cooperative Oncology Group performance status; ALP, alkaline phosphatase.

Four patients in the apatinib + AP group experienced recurrence or metastasis during the maintenance treatment with apatinib, leading to the discontinuation of apatinib treatment. The remaining 15 patients successfully completed a 1-year maintenance treatment.

### 3.2 Clinical effectiveness

Preoperative evaluation of the efficacy of neoadjuvant therapy revealed that 78.95% (15/19) and 73.91% (17/23) of patients in the apatinib + AP and MAP groups, respectively, experienced a reduction in ALP serum levels after neoadjuvant therapy (**Table 2**).

**Table 2:** Clinical outcomes of the two groups.

Characteristics	Apatinib + AP group (n = 19)	MAP group (n = 23)	P-value
Changes in ALP serum level post neoadjuvant therapy			1.000
Decreased	15 (78.95%)	17 (73.91%)	
Not decreased	4 (21.05%)	6 (26.09%)	
Response evaluated before surgery (RECIST)			0.852
PR	1 (5.26%)	1 (4.35%)	
SD	15 (78.95%)	17 (73.91%)	
PD	3 (15.79%)	5 (21.74%)	
Type of surgery			1.000
Limb salvage	17 (89.47%)	20 (86.96%)	
Amputation	2 (10.53%)	3 (13.04%)	
R0 resection			1.000
Yes	19 (100%)	23 (100%)	
No	0	0	
Tumor cell necrosis rate (%)			0.726
<90	4 (21.05%)	7 (30.43%)	
≥90	15 (78.95%)	16 (69.57%)	
M-DFS (months)	NA	16	0.183
1-year DFS rate (%)	78.9 (0.626−0.996)	59.4 (0.420−0.841)	0.165
2-year DFS rate (%)	61.5 (0.426−0.888)	44.5 (0.277−0.718)	0.283

Data are presented as numbers (percentages) or means ± standard deviations.

AP, doxorubicin-cisplatin chemotherapy; MAP, methotrexate-doxorubicin-cisplatin chemotherapy; ALP, alkaline phosphatase; RECIST, response evaluation criteria in solid tumors (version 1.1); PR, partial response; SD, stable disease; PD, progressive disease; M-DFS, median disease-free survival.

**Figure 1:**
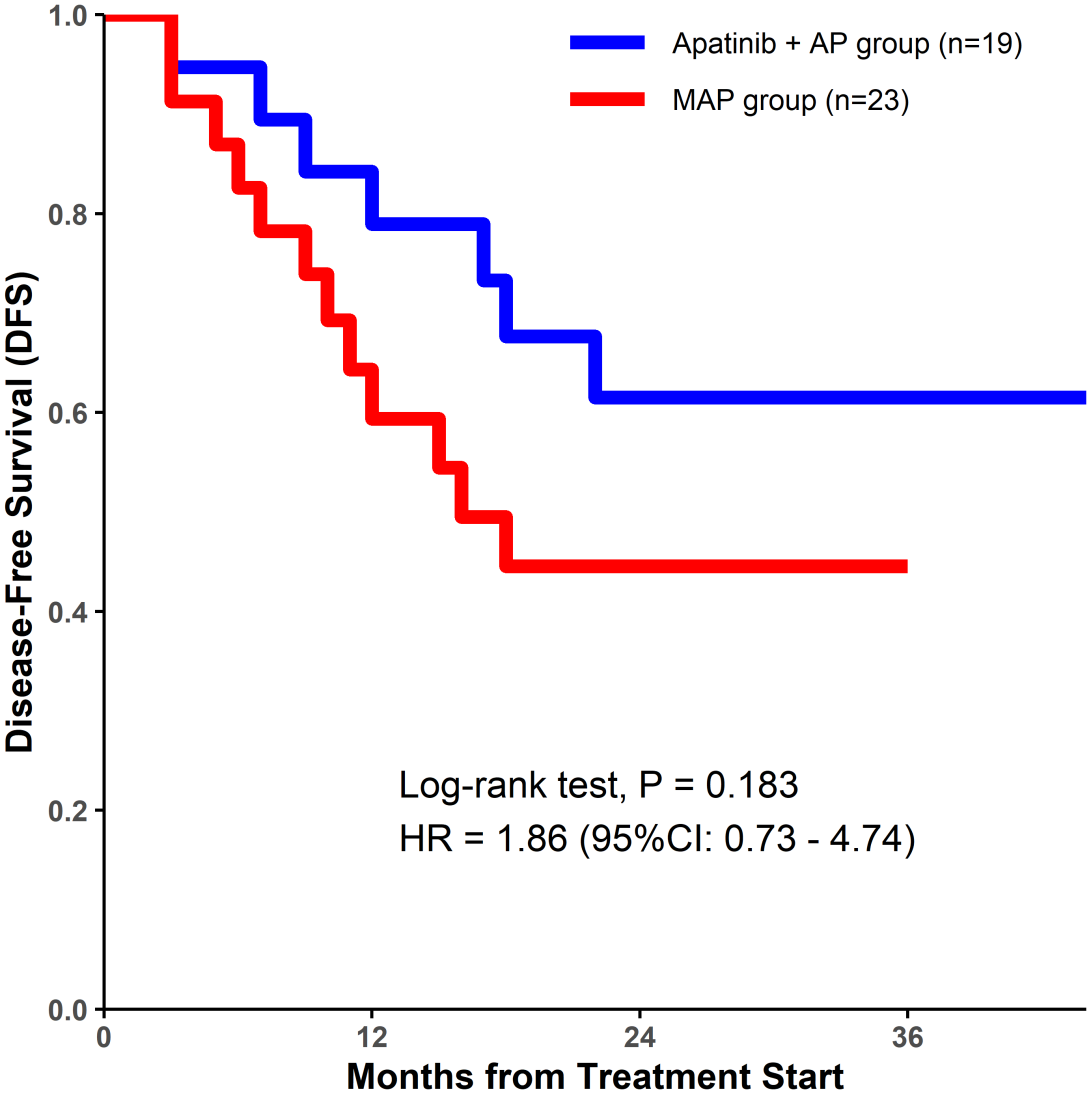
Kaplan–Meier estimates of disease-free survival for both treatment groups.

Although a decrease in ALP level was observed in a higher percentage of patients in the apatinib + AP group than in the MAP group, there were no significant differences between the two groups with respect to the DCR (84.21% vs. 78.26%, P=0.852; **Table 2**), tumor cell necrosis rate ≥90% (78.95% vs. 69.57%, P=0.726; **Table 2**), 1-year DFS rate (78.9% vs. 59.4%, P=0.165; **Table 2**, **Figure 1**), and the 2-year DFS rate (61.5% vs. 44.5%, P=0.283; **Table 2**, **Figure 1**).

### 3.3 Toxicity and complications

Patients in the apatinib + AP group experienced more AEs than those in the MAP group. Some AEs were significantly more common in the apatinib + AP group than in the MAP group (P<0.05), and these included oral mucositis (grades 3 and 4) (52.6% vs. 17.4%, respectively, P=0.023), limb edema (47.4% vs. 17.4%, respectively, P=0.049), hand-foot syndrome (31.6% vs. 0%, respectively, P=0.005), proteinuria (26.3% vs. 0%, respectively, P=0.014), hypertension (21.1% vs. 0%, respectively, P=0.035), and hypothyroidism (21.1% vs. 0%, respectively, P=0.035; **Table 3**).

**Table 3:** Neoadjuvant therapy-related adverse effects per treatment groups.

Characteristics	Apatinib + AP group (n = 19)		MAP group (n = 23)		P-value	
	All grades	Grade >2	All grades	Grade >2	All grades	Grade >2
Any toxicity	19 (100%)	19 (100%)	23 (100%)	21 (91.3%)	1.000	0.492
Leucopenia	19 (100%)	18 (94.7%)	23 (100%)	19 (82.6%)	1.000	0.356
Anaemia	18 (94.7%)	13 (68.4%)	21 (91.3%)	13 (56.5%)	1.000	0.530
Alopecia	18 (94.7%)	0 (0%)	21 (91.3%)	0 (0%)	1.000	1.000
Thrombocytopenia	17 (89.5%)	12 (63.2%)	15 (65.2%)	10 (43.5%)	0.083	0.232
Nausea	16 (84.2%)	11 (57.9%)	19 (82.6%)	9 (39.1%)	1.000	0.352
Oral mucositis	16 (84.2%)	10 (52.6%)	13 (56.5%)	4 (17.4%)	0.093	0.023
Fatigue	15 (78.9%)	5 (26.3%)	17 (73.9%)	2 (8.7%)	1.000	0.214
Anorexia	14 (73.7%)	5 (26.3%)	12 (52.2%)	2 (8.7%)	0.208	0.214
Transaminase increase	14 (73.7%)	4 (21.1%)	11 (47.8%)	1 (4.3%)	0.120	0.158
Vomiting	13 (68.4%)	4 (21.1%)	14 (60.9%)	3 (13.0%)	0.750	0.682
Fever	12 (63.2%)	2 (10.5%)	9 (39.1%)	1 (4.3%)	0.215	0.581
Diarrhoea	11 (57.9%)	2 (10.5%)	11 (47.8%)	1 (4.3%)	0.551	0.581
Pain	11 (57.9%)	0 (0%)	10 (43.5%)	1 (4.3%)	0.536	1.000
Limb edema	9 (47.4%)	2 (10.5%)	4 (17.4%)	0 (0%)	0.049	0.199
Weight loss	7 (36.8%)	0 (0%)	8 (34.8%)	0 (0%)	1.000	1.000
Constipation	6 (31.6%)	1 (5.3%)	7 (30.4%)	0 (0%)	1.000	0.452
Hand-foot syndrome	6 (31.6%)	1 (5.3%)	0 (0%)	0 (0%)	0.005	0.452
Proteinuria	5 (26.3%)	1 (5.3%)	0 (0%)	0 (0%)	0.014	0.452
Hypertension	4 (21.1%)	0 (0%)	0 (0%)	0 (0%)	0.035	1.000
Hypothyroidism	4 (21.1%)	0 (0%)	0 (0%)	0 (0%)	0.035	1.000
Dysgeusia	3 (15.8%)	0 (0%)	2 (8.7%)	0 (0%)	0.644	1.000
Cough	3 (15.8%)	0 (0%)	5 (21.7%)	0 (0%)	0.709	1.000
Pneumothorax	2 (10.5%)	1 (5.3%)	0 (0%)	0 (0%)	0.199	0.452

Data are presented as counts (percentages).

AP, doxorubicin-cisplatin chemotherapy; MAP, methotrexate-doxorubicin-cisplatin chemotherapy.

Postoperative complications in each group are shown in **Table 4**. A grade IV complication (cardiac failure) occurred in one patient in the MAP group (**Table 4**). There was no statistically significant difference in the incidence of postoperative complications between the two groups (P>0.05, **Table 4**). No drug- and surgery-related deaths occurred.

**Table 4:** Surgery-related complications per treatment group.

Complication	Apatinib +AP group (n = 19)	MAP group (n = 23)	P-value
Clavien-Dindo grading			0.912
Grade I	4	3	
Grade II	8	10	
Grade III	7	9	
Grade IV	0	1	
Grade V	0	0	
Wound infection	3 (15.8%)	4 (17.4%)	1.000
Pulmonary infection	2 (10.5%)	2 (8.7%)	1.000
Hemorrhage	2 (10.5%)	1 (4.3%)	0.581
Superficial wound dehiscence	7 (36.8%)	8 (34.8%)	1.000
Cardiac/respiratory failure	0	1 (3.85%)	1.000
The implant nonunion or fracture	5 (26.3%)	7 (30.4%)	1.000
Reoperation	7 (36.8%)	9 (39.1%)	1.000
Death	0	0	1.000

Data are presented as counts (percentages).

AP, doxorubicin-cisplatin chemotherapy; MAP, methotrexate-doxorubicin-cisplatin chemotherapy.

### 3.4 Univariate Cox regression analysis

**Figure 2:**
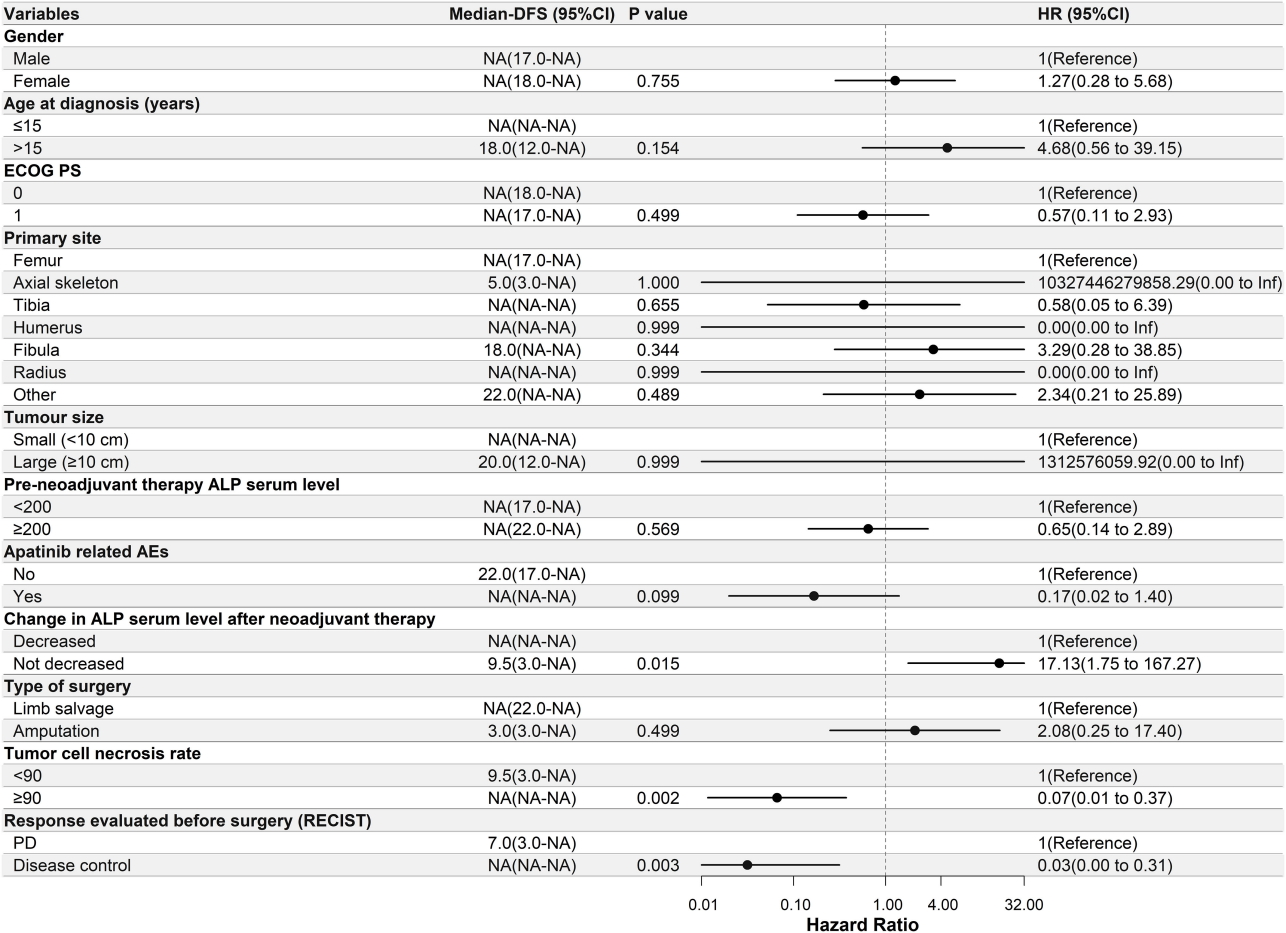
Univariate Cox regression analysis of the relationship between clinicopathological parameters and disease-free survival (DFS) in the apatinib + AP group. Patients with decreased alkaline phosphatase serum levels after neoadjuvant therapy, ≥90% tumor cell necrosis rate, and disease control had significantly longer DFS. ECOG PS, Eastern Cooperative Oncology Group performance status; ALP, alkaline phosphatase; AEs, adverse events; RECIST, Response Evaluation Criteria in Solid Tumors; PD, progressive disease; NA, Not Applicable.

**Figure 3:**
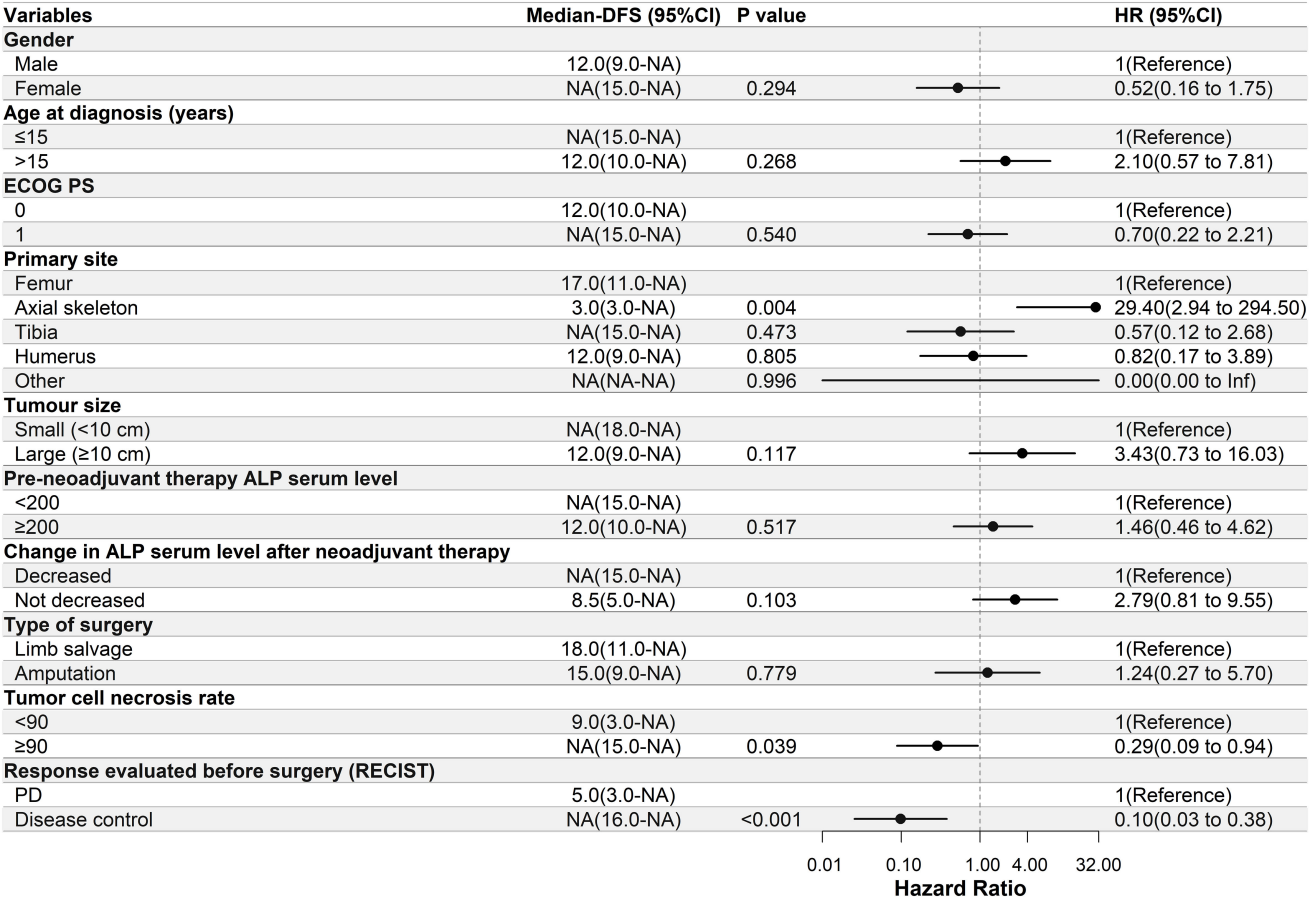
Univariate Cox regression analysis of the relationship between clinicopathological parameters and disease-free survival (DFS) in the methotrexate-doxorubicin-cisplatin group. Patients with a primary site located in the axial skeleton, ≥90% tumor cell necrosis rate, and disease control had significantly longer DFS. NA, Not Applicable. ECOG PS, Eastern Cooperative Oncology Group performance status; ALP, alkaline phosphatase; RECIST, Response Evaluation Criteria in Solid Tumors; PD, progressive disease; NA, Not Applicable.

Univariate Cox regression analysis was performed to determine the relationship between DFS and the clinical characteristics of the patients in this study. In the apatinib + AP group, patients with decreased ALP serum levels after neoadjuvant therapy, ≥90% tumor cell necrosis rate, and disease control after neoadjuvant therapy had significantly longer DFS (P<0.05, **Figure 2**). In the MAP group, patients with a primary tumor located in the axial skeleton, ≥90% tumor cell necrosis rate, and disease control after neoadjuvant therapy had significantly longer DFS (P<0.05, **Figure 3**).

## 4 Discussion

This study is the first to report the safety and effectiveness of apatinib plus AP for the neoadjuvant treatment of patients with osteosarcoma. Based on the different treatments received, patients were divided into the apatinib + AP and MAP groups. AEs were more prevalent in patients treated with apatinib plus AP than in those treated with MAP. The 1- and 2-year DFS rates in the apatinib +AP group were higher than those in the MAP group, but the difference was not significant.

Perioperative chemotherapy has repeatedly been shown to be indispensable as a standard of care for non-metastatic osteosarcoma (3, 7). However, chemotherapy drugs used remain controversial (8). The history of neoadjuvant chemotherapy in osteosarcoma is a balance between efficacy and toxicity, and researchers have tried to improve efficacy by increasing the dose or number of different drugs used as much as possible while maintaining a tolerable level of toxicity (3, 19). Currently, AP and MAP chemotherapies are generally recognized as efficacious, but they have a high toxicity rate, and their cure rate should be further improved (8). Improvements in neoadjuvant therapy for osteosarcoma will continue as new drugs are being developed (20–22).

Since it was launched, apatinib, a multi-target TKI, has been shown to have good efficacy in the treatment of advanced osteosarcoma that has failed multi-line therapy (23). Our previous study demonstrated that apatinib can reverse the resistance of osteosarcoma cells to doxorubicin (17). Different studies have also demonstrated inhibition of the targets of apatinib to be beneficial for the treatment of osteosarcoma (24). At present, multiple clinical trials of TKI combined with chemotherapy in the treatment of sarcoma have achieved benign results (25, 26). In the present study, the tumor necrosis rate and 1- and 2-year DFS rates in the apatinib +AP group were higher than those in the MAP group. This suggests that the addition of apatinib to neoadjuvant therapy for osteosarcoma could achieve better outcomes. This confirms the results of our previous study and similar studies (27, 28). However, the improvement in efficacy in the present study was not significant. We believe that the reason for this might be the limited number of patients. Therefore, it is necessary to further assess the efficacy of apatinib as neoadjuvant treatment of osteosarcoma in a prospective randomized controlled clinical trial with a large sample size.

Several previous studies have demonstrated that apatinib is highly toxic. This has led to a reduction in the dose used in clinical practice for the treatment of various malignancies from 750 mg to 500 mg (29, 30). Studies on various tumor types have demonstrated that apatinib combined with chemotherapy can be severely toxic (30, 31). This is an important reason apatinib has not been tested as neoadjuvant therapy for osteosarcoma, despite the evidence for efficacy in advanced sarcomas. In the present study, to reduce the toxicity of apatinib when combined with chemotherapy, the AP regimen was used for the combined group, which was also recommended as first-line treatment by the National Comprehensive Cancer Network guidelines (32). Nevertheless, the results of this study show that AEs were more prevalent in the apatinib + AP group than in the MAP group. However, these significantly increased adjuvant treatment-related AEs did not hinder the output of surgical treatment, let alone lead to adjuvant treatment-related death. This suggests that apatinib combined with AP in the neoadjuvant setting results in an acceptable level of toxicity. Here, we emphasize three points. First, in this study, the initial dose of apatinib was individualized according to the patient’s BSA, and the dose of apatinib was dynamically adjusted according to the occurrence of AEs. This is an important point for clinical decision-making and study design. Second, patients older than 30 years of age were excluded from receiving the combined regimen, which was fully considered from the outset of the study. Older patients have been excluded from several studies of neoadjuvant treatment in osteosarcoma (33). Younger patients appear to be more tolerant of the AEs caused by apatinib combined with chemotherapy. In addition, it is worth noting that in this study, seven patients younger than 10 years of age did not experience AEs when receiving apatinib combined with neoadjuvant chemotherapy. This suggests that apatinib can be safely added to neoadjuvant therapy in children with osteosarcoma. Finally, the maintenance treatment with apatinib after the MAP regimen is also a worthwhile treatment option. This approach not only avoids the toxicity of chemotherapy plus apatinib but also preserves the benefits of these two treatment options. The maintenance with apatinib after MAP may even be better than the apatinib + AP regimen.

Researchers have attempted to find the best evaluation system for neoadjuvant therapy for osteosarcoma (34–36). We found that decreased ALP serum levels after neoadjuvant therapy, ≥90% tumor cell necrosis rate, and disease control were significantly associated with longer DFS in the present study. This is similar to the results of other studies (12). However, it is unclear which method is the most suitable for efficacy evaluation and prediction of survival rates in patients treated with apatinib combined with AP neoadjuvant therapy. Answering this question requires further prospective studies and long-term follow-ups.

This study had some limitations, including its retrospective nature, small sample size, and short follow-up period. All these factors make it difficult to analyze the differences in outcomes and complications. Prospective registered clinical trials are required to continue investigating the effectiveness of the apatinib + AP regimen. Moreover, further research on the efficacy of apatinib in postoperative maintenance therapy for osteosarcoma is warranted.

## 5 Conclusions

The results of this study suggest that apatinib + AP may be a promising candidate for neoadjuvant therapy for osteosarcoma, warranting further validation in prospective randomized controlled clinical trials with long-term follow-up.

## Data availability statement

The original contributions presented in the study are included in the article/supplementary material. Further inquiries can be directed to the corresponding author.

## Ethics statement

The studies involving humans were approved by the Ethics Committee of Henan Cancer Hospital. The studies were conducted in accordance with the local legislation and institutional requirements. Written informed consent for participation in this study was provided by the participants’ legal guardians/next of kin.

## Author contributions

All authors made a significant contribution to the work reported, whether that is in the conception, study design, execution, acquisition of data, analysis and interpretation, or in all these areas; took part in drafting, revising or critically reviewing the article; gave final approval of the version to be published; have agreed on the journal to which the article has been submitted; and agree to be accountable for all aspects of the work.
